# Relationship between Auditory and Cognitive Abilities in Older Adults

**DOI:** 10.1371/journal.pone.0134330

**Published:** 2015-08-03

**Authors:** Stanley Sheft, Valeriy Shafiro, Emily Wang, Lisa L. Barnes, Raj C. Shah

**Affiliations:** 1 Department of Communication Disorders and Sciences, Rush University Medical Center, Chicago, Illinois, United States of America; 2 Rush Alzheimer’s Disease Center, Rush University Medical Center, Chicago, Illinois, United States of America; 3 Departments of Neurological Sciences and Behavioral Sciences, Rush University Medical Center, Chicago, Illinois, United States of America; Sun Yat-sen University, CHINA

## Abstract

**Objective:**

The objective was to evaluate the association of peripheral and central hearing abilities with cognitive function in older adults.

**Methods:**

Recruited from epidemiological studies of aging and cognition at the Rush Alzheimer’s Disease Center, participants were a community-dwelling cohort of older adults (range 63–98 years) without diagnosis of dementia. The cohort contained roughly equal numbers of Black (n=61) and White (n=63) subjects with groups similar in terms of age, gender, and years of education. Auditory abilities were measured with pure-tone audiometry, speech-in-noise perception, and discrimination thresholds for both static and dynamic spectral patterns. Cognitive performance was evaluated with a 12-test battery assessing episodic, semantic, and working memory, perceptual speed, and visuospatial abilities.

**Results:**

Among the auditory measures, only the static and dynamic spectral-pattern discrimination thresholds were associated with cognitive performance in a regression model that included the demographic covariates race, age, gender, and years of education. Subsequent analysis indicated substantial shared variance among the covariates race and both measures of spectral-pattern discrimination in accounting for cognitive performance. Among cognitive measures, working memory and visuospatial abilities showed the strongest interrelationship to spectral-pattern discrimination performance.

**Conclusions:**

For a cohort of older adults without diagnosis of dementia, neither hearing thresholds nor speech-in-noise ability showed significant association with a summary measure of global cognition. In contrast, the two auditory metrics of spectral-pattern discrimination ability significantly contributed to a regression model prediction of cognitive performance, demonstrating association of central auditory ability to cognitive status using auditory metrics that avoided the confounding effect of speech materials.

## Introduction

Aging is characterized by both sensory and cognitive decline. Significant association between hearing loss, as measured by detection thresholds for pure-tone stimuli, and cognitive function has been reported in some [1–5] but not all studies of older adults [6–11]. Across studies, differences in the cognitive status and age range of the study participants, audiometric testing environment, cognitive measures used, and statistical control of demographic variables most likely contributed to the difference in results. A variety of factors have been proposed as a basis for the relationship between hearing acuity and cognitive performance. Termed the common-cause hypothesis, Lindenberger and Bates [2] speculated that the association could arise from an age-related decline in the physiological integrity of neural subsystems common to both sensory and cognitive processing. With a common cause, sensory and cognitive aging would occur concurrently. Alternatively, Uhlmann et al. [1] proposed that sensory deprivation due to hearing loss may contribute to a subsequent decline in cognitive function. Support for involvement of a cascading consequence of hearing loss comes from the work of Peelle et al. [12] who, in an fMRI study, found a relationship between speech abilities and cortical structure and function. The authors suggested that peripheral hearing loss may lead to a systematic breakdown in the regulation of neural activity and a loss of gray matter. Along with physiological effect, hearing loss may either degrade the sensory information needed for proper cognitive function [13] or affect the allocation of limited cognitive resources [4,5,12]. More broadly, a hearing loss may lead to a reduction in the extent of social interaction with an associated effect on cognitive status [1,5]. Finally, in contrast to suggestions of interaction, involvement of independent factors may lead to parallel aging in terms of hearing acuity and cognitive function [7].

Change in hearing sensitivity associated with aging primarily reflects the physiologic integrity of the peripheral hearing system. Central presbycusis refers to age-related change in the auditory nervous system beyond the periphery [14]. Behavioral measures of central auditory processing commonly assess perception of low-redundancy or distorted speech, often with competing signals or dichotic presentation, and psychoacoustic abilities involving temporal patterns and binaural interaction. Investigation in older adults of the relationship between cognitive mechanisms and peripheral and central auditory processing has focused on two main areas of study. In one, the motivation has been to better understand the involvement and interaction of factors that determine the decline in speech abilities with aging (see [15–16] for reviews). The other concern has been with the relationship between the integrity of auditory function and cognitive status. In studies with older adults, results have demonstrated an association between poorer performance on tests of central auditory processing and a diagnosis of either Alzheimer’s disease (AD) or mild cognitive impairment (MCI) [6,10,17]. Importantly, this finding has been obtained despite control in terms of peripheral auditory function [10,18,19]. Reviewing published literature on the relationship between tests of central auditory processing disorders (CAPD) and AD, Iliadou and Kaprinis [20] concluded that CAPD tests may show an early manifestation of AD, proceeding clinical diagnosis by 5–10 years. Gates et al. [21] concluded that central auditory dysfunction is a precursor to AD, and furthermore suggested that CAPD tests may help predict the risk of a later diagnosis of AD.

Evidence from research using speech perception tests and CAPD test batteries with MCI and AD patients is promising in that it consistently confirms a relationship between central auditory processing abilities and cognitive function in dementia, even after controlling for the effects of hearing acuity. However, administering a complete CAPD battery is a lengthy process, which often may not be clinically feasible, while individual tests from the battery may be considerably less sensitive to cognitive function. Furthermore, CAPD tests require both extensive tester training and explicit use of a specific language. Some CAPD tests also exhibit diminished predictive power due to basic hearing deficits such as hearing loss. Finally, most of the CAPD tests use speech stimuli or require verbal response. Interpretation of the association between auditory and cognitive performance results is therefore confounded by cognitive involvement in auditory measures reliant on speech processing. This concern led Humes et al. [14] to argue for the development of tests of central presbycusis with greater auditory specificity.

Avoiding speech materials, Sheft et al. [22] recently introduced brief psychoacoustic procedures for evaluating auditory discrimination abilities for both static and dynamic spectral patterns. For the static spectral patterns, the test measured thresholds for discriminating a change in the phase of a low-rate sinusoidal spectral ripple of wideband noise, evaluating the ability to discriminate change in either the timbre or pitch of a broadband stimulus. Discrimination of dynamic spectral patterns was assessed as the threshold signal-to-noise ratio (SNR) needed to discriminate low-rate stochastic patterns of frequency modulation (FM) of a tonal carrier. With the noise modulators drawn from a single sampling distribution, the condition assessed ability to discriminate between patterns of frequency fluctuation for stimuli that shared common characteristics, notably average modulation rate and maximum frequency excursion. Results from adult listeners in the study of Sheft et al. [22] showed an effect of aging and a relationship with the perception of distorted speech and speech in noise. Thus, measurement of spectral-pattern discrimination offers a potential way to evaluate the relationship between central auditory processing and cognitive function that avoids the confounding effect of use of speech materials in the auditory testing.

The aim of the current study was to assess the associations between spectral-pattern discrimination abilities and performance on a cognitive test battery in older adults. Along with discrimination thresholds, measures of hearing sensitivity and speech-in-noise perception were considered in the modeling. Previous research used participants diagnosed with either AD or MCI to evaluate the association between central auditory processing and cognitive status. These disorders may introduce general performance factors which influence assessment of relationships among metrics. For the current study, participants were a community-dwelling cohort of older adults without diagnosis of dementia. Past work has documented racial differences in the cognitive scores of Black and White older adults [23–24]. Reasons for the disparities are not completely understood but minority status is associated with different life experiences and environmental exposures that can complicate assessment of cognition. Older African Americans are more likely to have both poorer quality and fewer years of formal education, fewer socioeconomic resources, and poorer health, factors that have been shown in numerous studies to be associated with poorer performance on many measures of cognitive function [25–28]. On the other hand, past research has also revealed superior performance of older Black than White adults in terms of hearing sensitivity [29–30]. We are aware of no work that has evaluated racial differences on auditory measures using more complex stimuli so that results may relate more closely to central auditory processing. Therefore, a secondary aim of the current study was to compare the auditory abilities—including the measures of spectral-pattern discrimination—of Black and White older adults in relation to their cognitive function. Roughly equal numbers of Black and White participants were recruited with groups similar in terms of age, gender, and years of education. The participants were recruited from ongoing studies at the Rush University Medical Center with results intended to provide a baseline for longitudinal investigation of auditory abilities as predictors of cognitive decline.

## Methods

### Ethics statement

All methods were approved by the Institutional Review Board of the Rush University Medical Center, and all participants provided written informed consent.

### Subjects

Each of the 124 subjects was enrolled in one of two epidemiologic studies of aging and cognition at the Rush Alzheimer’s Disease Center. All were individuals without known dementia. The 61 self-identified Black participants were recruited from the Rush Alzheimer’s Disease Clinical Core [31], while the 63 self-identified White participants were from the Memory and Aging Project (MAP [32]). Participants for both studies were recruited from the community. In their majority, MAP participants are typically residents in continuing care retirement communities, senior subsidized housing, or individual homes. Participants in the Clinical Core are recruited through churches, senior organizations, and senior subsidized housing. The recruitment techniques, data collection, and study operations are similar between the studies.

Demographic information with statistical analysis is summarized in Table 1. Results indicate a close match between Black and White participants included in the study sample. For the entire cohort of 124 subjects, age ranged from 63 to 98 years with a mean (SD) age of 74.6 (5.7) and 75.9 (6.9) years for the Black and White participants, respectively. Both subject groups were composed primarily of women (88–89%) with almost identical mean number of years of education. The Mini-Mental State Examination [33] was also used to describe the cohort. Using *t* tests for continuous variables and Pearson’s χ^2^ for the categorical variable gender, there were no significant differences between the Black and White subject groups in terms of demographic variables or Mini-Mental performance.

**Table 1:** Demographic characteristics.

	Black (*n* = 61)	White (*n* = 63)	*t* value (*df)*	*p* value	Cohen’s *d*
**Age (years)**	74.6 (5.7)	75.9 (6.9)	0.26 (122)	.267	.20
**Education (years)**	15.5 (3.6)	15.3 (3.2)	-0.21 (122)	.735	-.03
**Women (%)**	89	88		.835	
**Mini-Mental State Exam (score)**	28.2 (1.7)	28.7 (1.6)	1.76 (122)	.080	.32

Values are arithmetic mean (SD), except for women in percent. *t* values, degrees of freedom (*df*), and *p* values are from *t* tests of differences between Black and White subjects, except for percent women which was evaluated with Pearson’s χ^2^. Cohen’s *d* is a measure of effect size for each test.

During a single session, subjects participated in cognitive and auditory testing.

### Cognitive measures

Cognitive function was evaluated with a battery of 12 tests, requiring approximately one hour. Based on past work [34–36], the 12 tests were categorized as assessments of five cognitive domains: episodic, semantic, and working memory, perceptual speed, and visuospatial abilities. In this past work, principal-components factor analysis with a varimax rotation was used for empirical grouping of tests. Tests with a rotated factor loading of 0.5 or higher were grouped on a common factor. Tests that loaded on more than one factor were assigned according to the highest loading. Using Rand’s statistic [37], validity of these empirical groupings was confirmed by comparison to conceptually based division of the tests into specific functional domains. Subsequent evaluation using all possible permutations of test groupings established that the agreement between empirical and conceptual groupings could not be achieved by chance alone.

The 12 tests included five measures of episodic memory (immediate and delayed recall of Story A from the Logical Memory subtest of the Revised Wechsler Memory Scale [38]; Word List Memory, Recall, and Recognition [39]), one test of perceptual speed (Symbol Digit Modalities Test [40]), two tests of semantic memory (Category Fluency and a 15-item version of the Boston Naming Test [39]), two tests of visuospatial abilities (a 15-item version of Judgment of Line Orientation [41]; a 16-item version of Standard Progressive Matrices [42]), and two measures of working memory (Digit Span forward and backward from the Revised Wechsler Memory Scale [38]). Using the mean and standard deviations from baseline cognitive testing of the entire combined cohort of the Clinical Core (n = 289) and MAP (n = 1631) studies, performance on individual tests was converted to a *z* score with subsets of *z* scores averaged to obtain measures of domain-specific performance. All *z* scores were averaged as a measure of global cognition. In all cases, higher *z* scores indicate better performance.

### Auditory measures

There were four metrics of auditory ability, an audiogram, a measure of speech-in-noise ability, and two measures of spectral-pattern discrimination.

#### Audiogram

The first test, an audiogram, assessed hearing sensitivity in each ear at the octave frequencies between 500 and 4000 Hz. For analysis, audiometric results were summarized as the speech-frequency pure-tone average (PTA), the average of the four hearing thresholds calculated for the better-hearing ear.

#### Speech-in-noise intelligibility

The second auditory metric evaluated speech perception in terms of the intelligibility of sentences from the Quick Speech-in-Noise Test (QuickSIN) in the presence of a four-talker speech-babble masker [43]. Each QuickSIN list contains six sentences with SNR decreasing in 5-dB steps from 25 to 0 dB across sentences. Based on the number of key words correctly repeated, results were converted to the metric SNR Loss, the estimated SNR needed for 50% correct relative to the performance of normal-hearing young adults. This metric thus represents a normalized speech reception threshold. Two scored lists were used along with a single practice list. QuickSIN testing was conducted with diotic presentation. Following the clinical protocol recommended by the test developer, presentation level was determined by the subject’s PTA. If the average of the pure-tone thresholds at 500, 1000, and 2000 Hz was 45 dB HL or less, the QuickSIN presentation level was 70 dB HL. Otherwise, the presentation level was at what the subject judged as loud but still a comfortable listening level. Of the 124 study participants, this second method of setting level was used with two subjects.

#### Spectral-pattern discrimination

**Fig 1:**
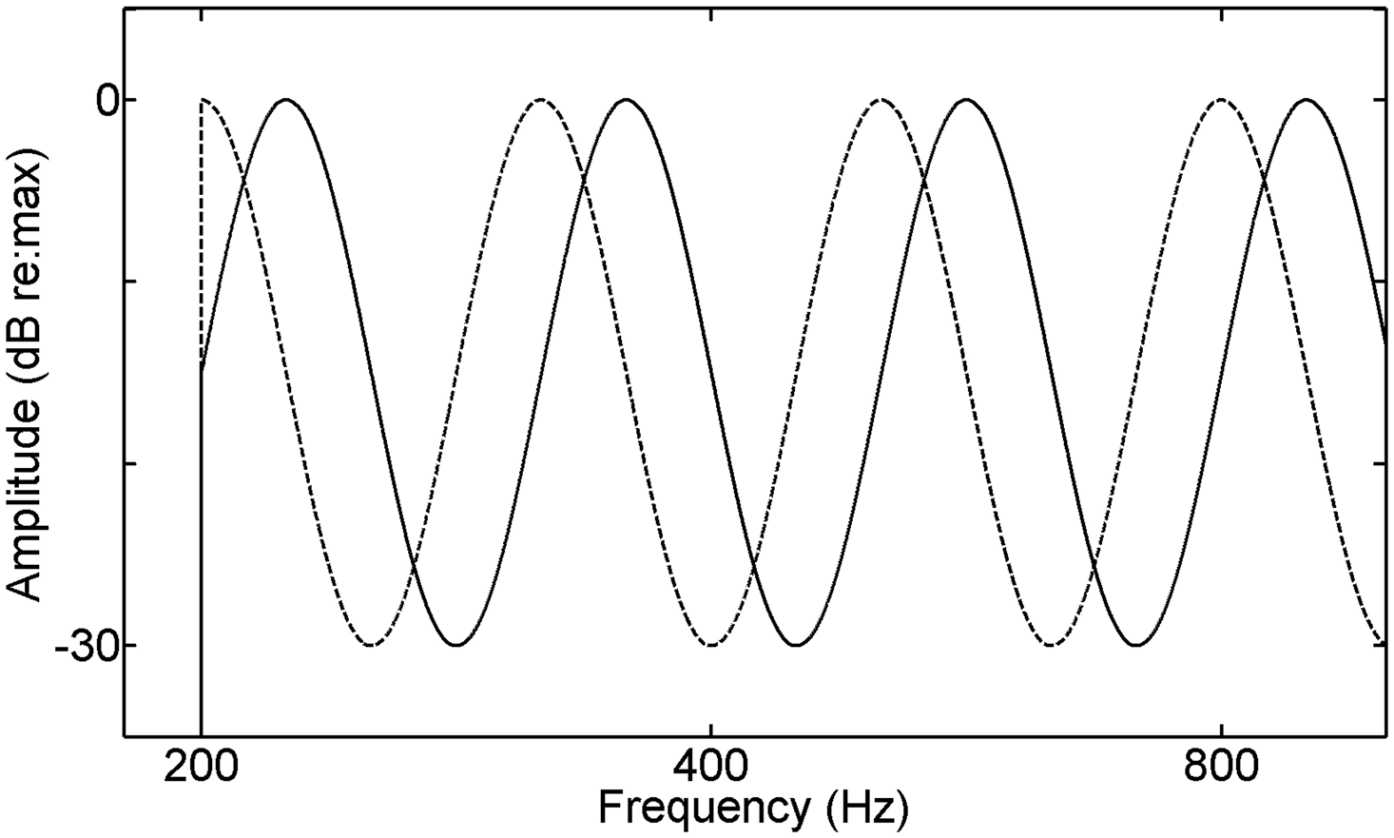
Ripple-phase discrimination. Schematic illustration of the spectral-ripple condition showing the contrasting amplitude spectra of a discrimination trial with difference due to change in starting phase of the spectral ripple. The speech-shaped filtering of the stimuli is omitted in the illustration.

The final two auditory metrics assessed discrimination ability for either static or dynamic spectral patterns. In the first, the patterns were logarithmically scaled sinusoidal ripples of the long-term amplitude spectrum of wideband noise stimuli. Consequently, the spectral patterns were static (constant) over the stimulus duration. Derived from noise, the stimuli contained rapid fluctuations of both envelope and fine structure. The temporal patterns of these fluctuations were random and varied across the noise samples used in stimulus generation. To generate stimuli, the amplitude spectra of samples of wideband noise were sinusoidally rippled in terms of the logarithms of both frequency and amplitude. Ripple density was 1.5 cycles per octave with a peak-to-trough difference of 30 dB. The task measured the just-detectable change in the starting phase of the spectral ripple with randomization of ripple phase across trials (Fig 1). The 500-ms rippled stimuli were shaped with a 50-ms rise/fall time, passed through a speech-shaped filter, and presented diotically at 80 dB SPL. Based on the findings of Byrne et al. [44], the speech filter emphasized the mid frequencies (roughly 200–500 Hz), with a steep roll-off in the low frequencies of over 20-dB per octave and a gradual roll-off of roughly 3- to 6-dB per octave in the high frequencies. Ripple-phase thresholds in radians were logarithmically transformed before statistical analysis.

**Fig 2:**
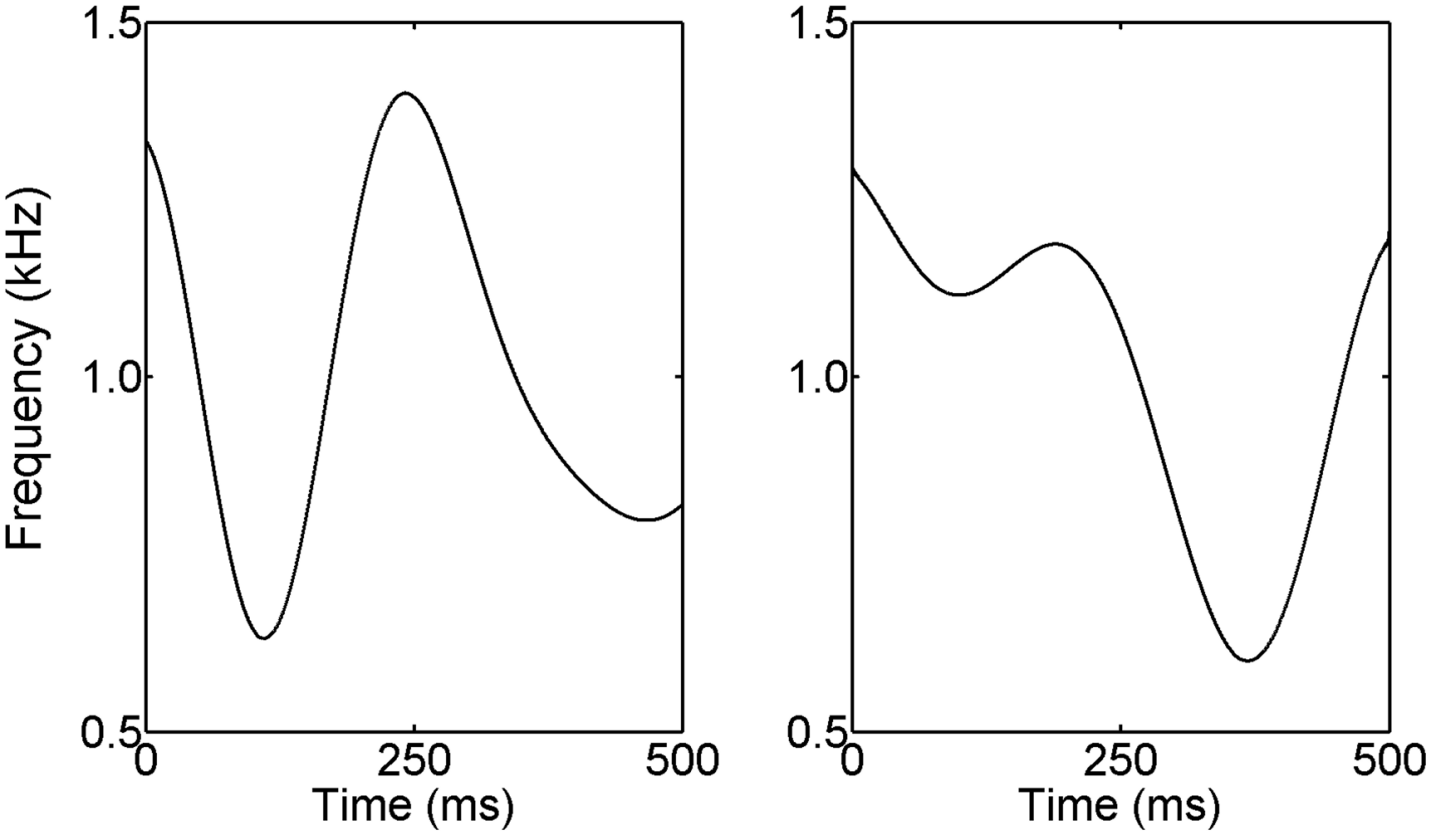
Stochastic FM discrimination. Schematic illustration of stochastic FM showing the contrasting instantaneous frequency functions of two stimuli of a discrimination trial.

**Fig 3:**
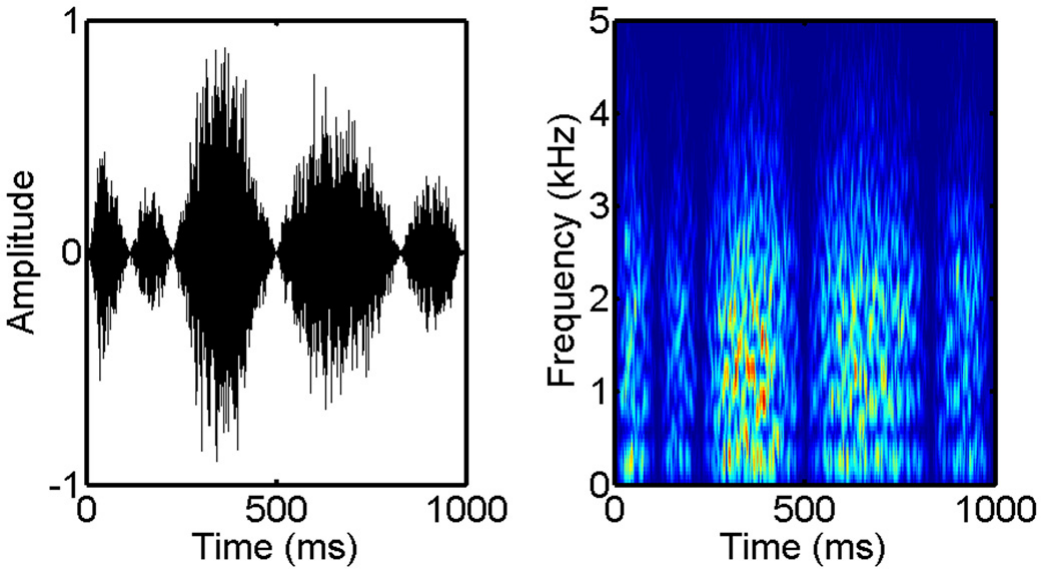
Masker from FM SNR condition. Left panel: time waveform of a masker sample used in the FM SNR condition. Right panel: spectrogram of the masker sample showing the amplitude spectrum as a function of time.

The final auditory condition utilized FM to generate dynamic spectral patterns whose spectral content varied over time. The procedure evaluated the ability to discriminate 1-kHz pure tones, frequency modulated by different samples of 5-Hz lowpass noise. A consequence of the modulation is that the instantaneous frequency of the stimulus follows the slowly fluctuating amplitude pattern of the 5-Hz noise modulator. The maximum frequency excursion (ΔF) was fixed at 400 Hz. With ΔF fixed and a common sampling distribution of noise modulators, discrimination can rely on only the temporal pattern of frequency deviation (Fig 2). The 500-ms stimuli were temporally centered in a 1000-ms masker with thresholds measured in terms of the SNR needed to just discriminate the pattern of frequency fluctuation. To have modulation characteristics similar to speech but without the confounding effect of speech content, maskers were speech-shaped wideband noise processed to include slow random variations in fine-structure periodicities and loudness. The fine-structure periodicities were introduced through an iterative delay-add process in which delay time was dynamically varied between 0.75–3.0 ms by the time structure of 15-Hz lowpass noise. The loudness variations were achieved by comodulating the maskers with 2.5-Hz lowpass noise. The temporal waveform and spectrogram of a masker sample are shown in Fig 3. Signals and maskers were separately shaped with a 50-ms rise/fall time. In the diotic listening task, masker level was fixed at 80 dB SPL with the level of the FM tones varied to estimate the threshold SNR.

A cued two-interval forced-choice (2IFC) procedure with three stimulus presentations per trial was used in both the ripple-phase and FM SNR conditions. In the procedure, the cue was the second stimulus presentation with subjects verbally indicating their selection of which observation interval differed from the cue. Using a modified descending method of limits, thresholds were derived from performance on a single 42-trial block, cycling seven times from high to low through six levels of the independent variable of a given condition, either delta ripple phase or FM SNR. For the ripple-phase condition, the starting delta was 2.3 radians with each subsequent delta smaller by a factor of 0.54. In the FM condition, the six values of SNR ranged from -18 to 12 dB with 6 dB between adjacent levels.

The 2IFC psychometric function ranges between 50 and 100% correct. Assuming a stable underlying function with function slope symmetric about threshold at 75% correct, threshold can be arithmetically derived if the levels of the independent variable are evenly spaced and at least minimally bracket the threshold point. Specifically, threshold is:

high+step/2–step*(2*p-num),

where *high* is highest level of the independent variable, *step* is the decrement between successive levels of the independent variable, *num* is the number of levels used, and *p* is the sum of the correct-response probabilities across all levels. A final assumption used in threshold derivation is that no response probability can be below chance performance. In the ripple-phase condition, logarithmic values of the variables *high* and *step* were used in threshold estimation, while values in the FM condition were from SNRs in dB. Preceded by a 12-trial practice block, a single threshold estimate was derived for each listener in each condition.

Auditory testing was conducted in a quiet room at the participant’s home or residence. Laptop computers with Sennheiser HD 280 Pro headphones were used for stimulus presentation. Systems were calibrated by measuring sound levels through a Knowles Electronic Manikin for Acoustic Research.

### Statistical analysis

Data were analyzed to i), evaluate the relationship between auditory and cognitive abilities and ii), examine potential differences between participants based on race. First, demographic and test performance differences between Black and White subject groups were evaluated. Next, a linear regression model was used to evaluate the association between global cognitive performance and auditory covariates, controlling for demographic variables. To further detail aspects of cognitive function related to auditory performance, commonality analysis [45] was used to partition the regression effect. This analysis included all possible combinations of auditory and demographic factors to determine the unique and shared variance contributions of each covariate to the model. Finally, auditory and domain-specific cognitive covariates were submitted to a factor analysis to assess the interrelationships among variables, with regression modeling repeated for the domain-specific cognitive variable working memory.

## Results

### Cognitive performance

**Fig 4:**
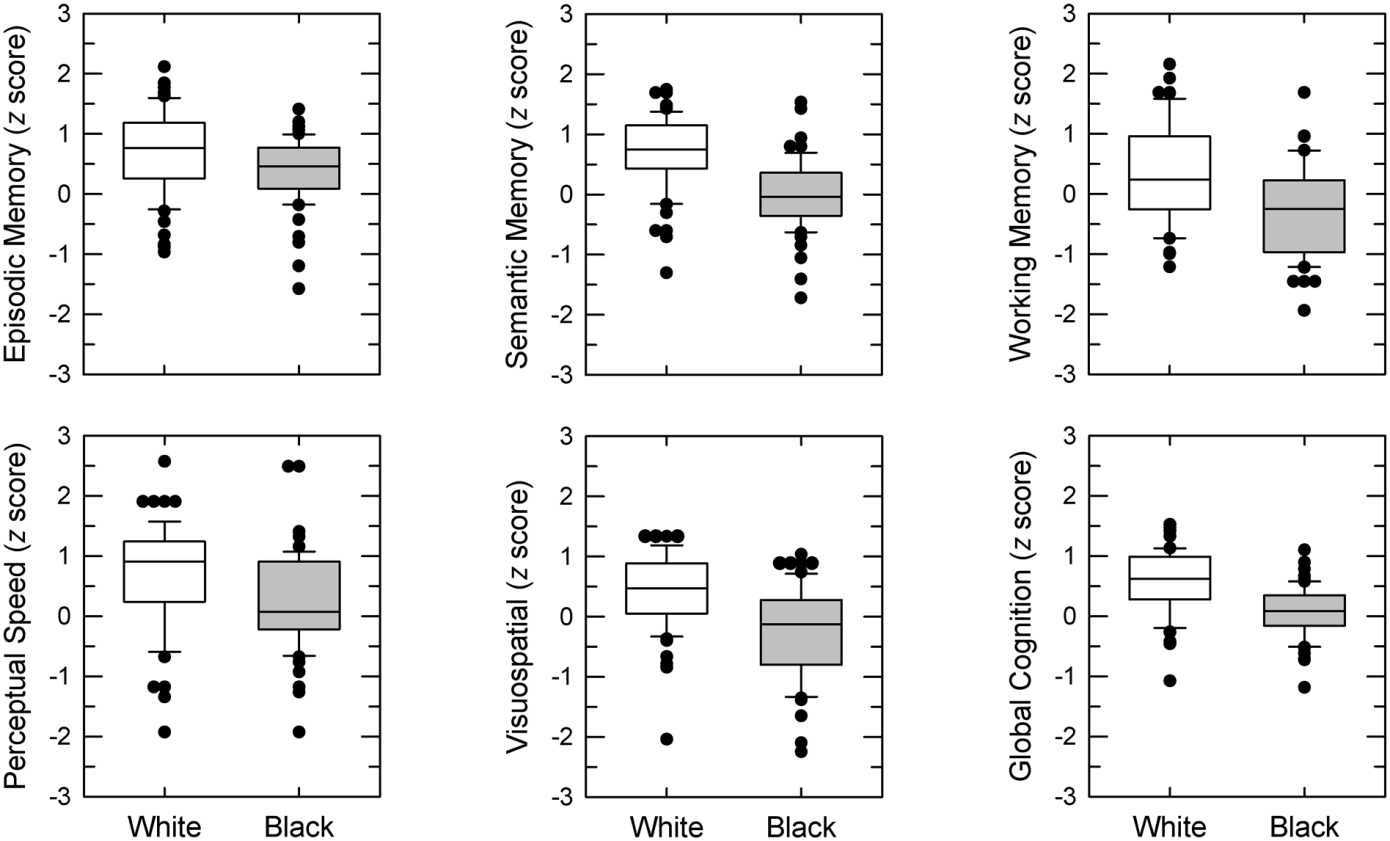
Cognitive test results. In separate panels for the five domain-specific cognitive metrics and global cognition, results as z scores for each subject group (White or Black). Error bars show the 10th and 90th percentiles, with outliers exceeding the error-bar range plotted as filled circles.

Mean group performance, along with statistical outcomes, of cognitive testing are in the top section of Table 2. Box plots of the data are shown in Fig 4. The mean score on the composite measure of global cognition was one half of a standard unit lower for Black than White subjects, and the scores on the five specific cognitive domains ranged from about one third standard unit lower in episodic memory to roughly two thirds of a standard unit lower for semantic and working memory and visuospatial ability. Significant between-group differences were obtained on all normalized measures of cognitive function with better performance by White than Black subjects.

**Table 2:** Test results.

	Black (*n* = 61)	White (*n* = 63)	*t* value (*df*)	*p* value	Cohen’s *d*
**Cognitive Test Result**					
**Global cognition (*z* score)**	0.081 (0.420)	0.582 (0.520)	5.90 (122)	>.001	1.06
**Episodic memory (*z* score)**	0.372 (0.547)	0.685 (0.684)	2.81 (122)	.018	0.51
**Semantic memory (*z* score)**	0.008 (0.591)	0.677 (0.619)	6.15 (122)	>.001	1.11
**Working memory (*z* score)**	-0.335 (0.733)	0.330 (0.825)	4.73 (122)	>.001	0.85
**Perceptual speed (*z* score)**	0.218 (0.802)	0.682 (0.887)	3.05 (122)	.012	0.55
**Visuospatial (*z* score)**	-0.226 (0.741)	0.436 (0.622)	5.40 (122)	>.001	0.97
** Auditory Test Result**					
**Speech-frequency PTA (dB)**	21.6 (9.1)	24.1 (12.0)	1.29 (115.2)	.396	0.23
**QuickSIN SNR Loss (dB)**	3.2 (2.8)	4.2 (5.7)	1.23 (89.7)	.396	0.22
**Ripple-phase threshold (radians)**	0.63 (1.89)	0.39 (1.69)	-4.59 (116.2)	>.001	0.82
**FM SNR threshold (dB)**	2.8 (6.3)	-1.2 (6.8)	-3.43 (122)	.005	0.62

Values are arithmetic mean (SD), except for ripple-phase threshold with the geometric mean (SD) reported. *t* values, degrees of freedom (*df*), and *p* values are from *t* tests of differences between Black and White subjects with the Bonferroni-Holm correction for multiple comparisons. When less than 122, *df* was adjusted due to significance of Levene’s test for equality of variances. Cohen’s *d* is a measure of effect size for each test.

### Auditory abilities

**Fig 5:**
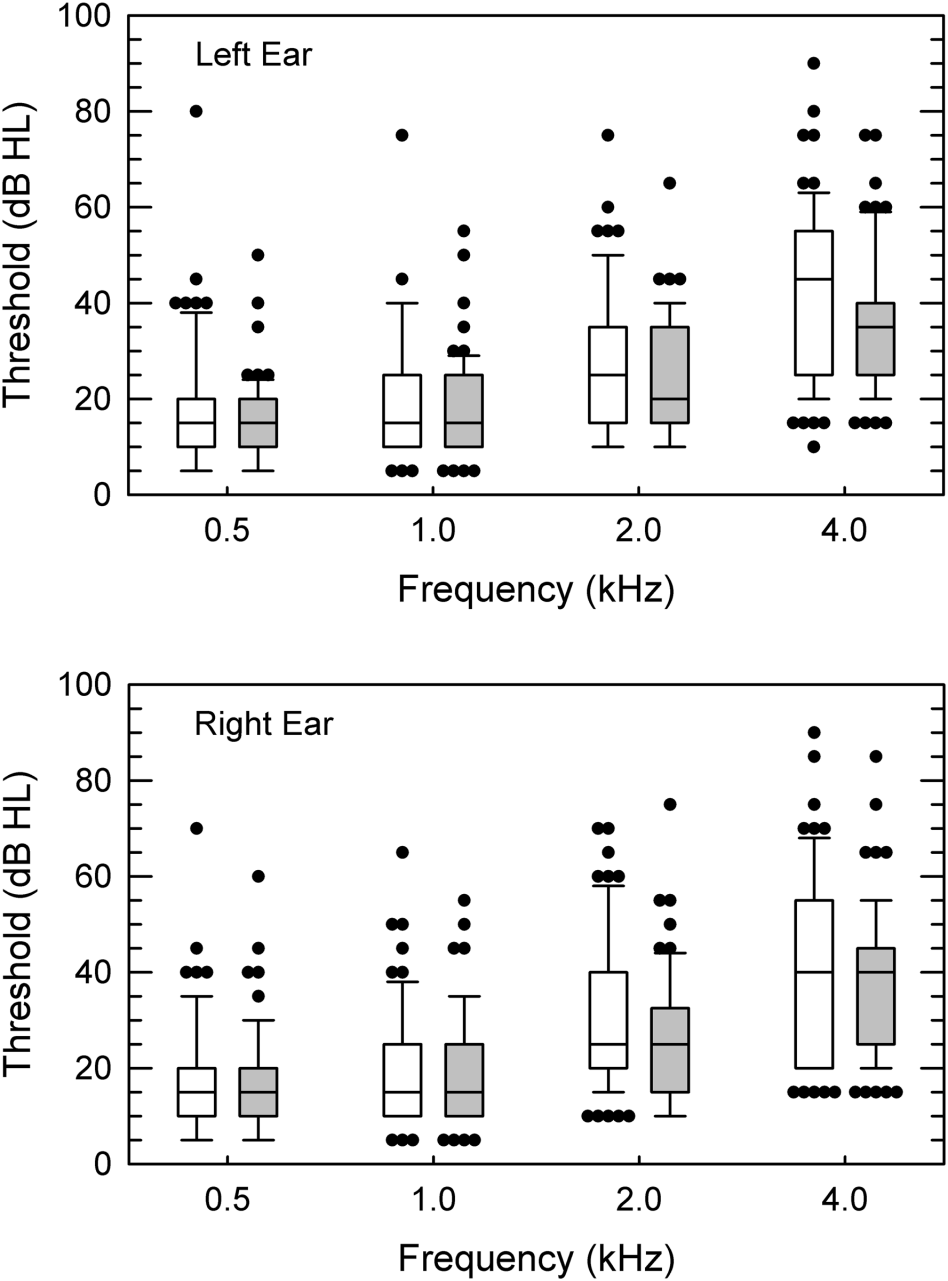
Audiograms. In separate panels for the left and right ear, group audiograms. Subject group is indicated by box shading with the White group without shading and the Black group with gray shading. Error bars show the 10th and 90th percentiles, with outliers exceeding the error-bar range plotted as filled circles.

**Fig 6:**
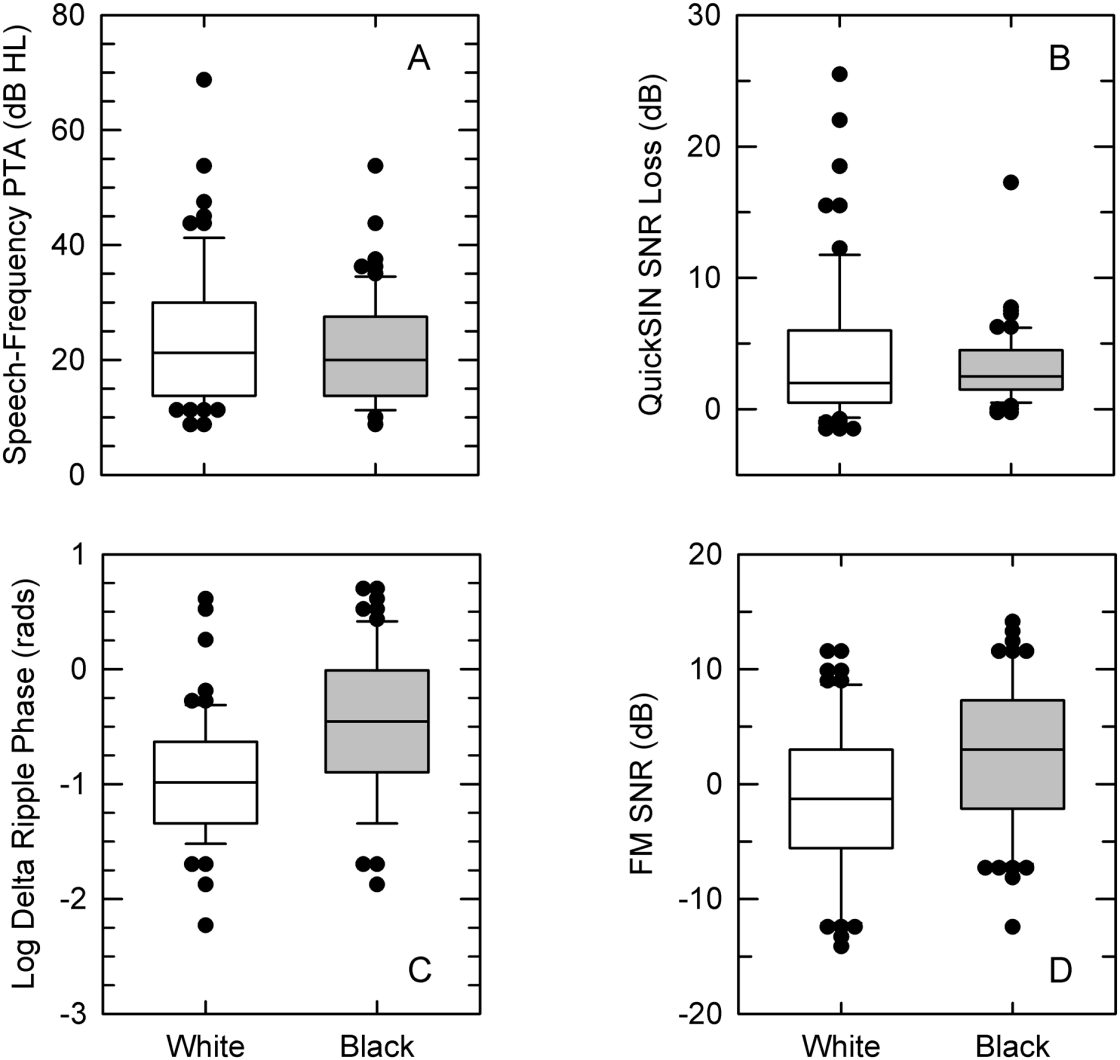
Auditory test results. In separate panels for each of the four auditory metrics, results from each subject group (White or Black). Error bars show the 10th and 90th percentiles, with outliers exceeding the error-bar range plotted as filled circles.

Mean group performance, along with statistical outcomes, for the four auditory measures are in the bottom section of Table 2. Group audiograms for each ear are shown in Fig 5. Most subjects exhibited relatively symmetric between-ear hearing sensitivity. For 98 of the 124 subjects, their right- and left-ear speech-frequency PTAs differed by 5 dB or less, with an additional seven subjects showing a difference of less than 10 dB. Only two subjects had a PTA difference greater than 25 dB, with values of 37.5 and 50 dB. Classifying normal hearing as a PTA ≤ 25 dB, a mild loss as > 25 dB and ≤ 40 dB, and a moderate loss as > 40 dB and ≤ 70 dB, results from both subjects indicated normal hearing in one ear and a moderate hearing loss in the other. Across all subjects, hearing sensitivity, as assessed by their better-ear speech-frequency PTA, ranged from 8.75 to 68.75 dB HL, with regression analysis indicating a 0.9-dB increase in PTA per year of subject age. Based on these PTAs, roughly 62% of subjects exhibited normal hearing, 31.5% showed a mild loss, and 6.5% a moderate hearing loss. On average, the PTA of Black subjects was 2.5-dB lower than that of White subjects (Fig 6, panel A). However neither PTA (see Table 2) nor breakdown by hearing-loss classification [χ^2^ (2, *N* = 124) = 2.52, *p* = .283] was significantly different between the two groups.

Results from speech-in-noise testing as assessed by QuickSIN are shown in panel B of Fig 6. Across all subjects, QuickSIN SNR Loss thresholds spanned a range of 27 dB, with mean thresholds of 3.2 and 4.2 dB for the Black and White subject groups, respectively. One-sample *t* tests were used to compare group performance to 0 dB, the normalized QuickSIN SNR Loss for normal-hearing young adults [43]. For both Black [*t*(60) = 9.11, *p* < .001, *d* = 1.17] and White [*t*(62) = 5.80, *p* < .001, *d* = 0.73] groups, the comparisons were significant. However, the performance difference between the Black and White subject groups was not significant (see Table 2).

Thresholds from the ripple-phase and FM SNR conditions for each group are displayed in panels C and D, respectively, of Fig 6. In the figure, as well as in statistical analysis, ripple-phase thresholds in radians were logarithmically transformed. Without the transformation, individual ripple-phase thresholds ranged from 0.11 to 2.01 radians, with mean thresholds of 0.63 and 0.39 radians for the Black and White subject groups, respectively. Across all subjects in the FM SNR condition, thresholds spanned a range of slightly over 28 dB, with mean thresholds of 2.8 and -1.2 dB for the Black and White subject groups, respectively. In contrast to hearing sensitivity and speech-in-noise ability, significant group differences were obtained in the ripple-phase and FM SNR conditions with better performance by White than Black subjects (see Table 2).

### Relationship between global cognitive performance and auditory abilities

**Fig 7:**
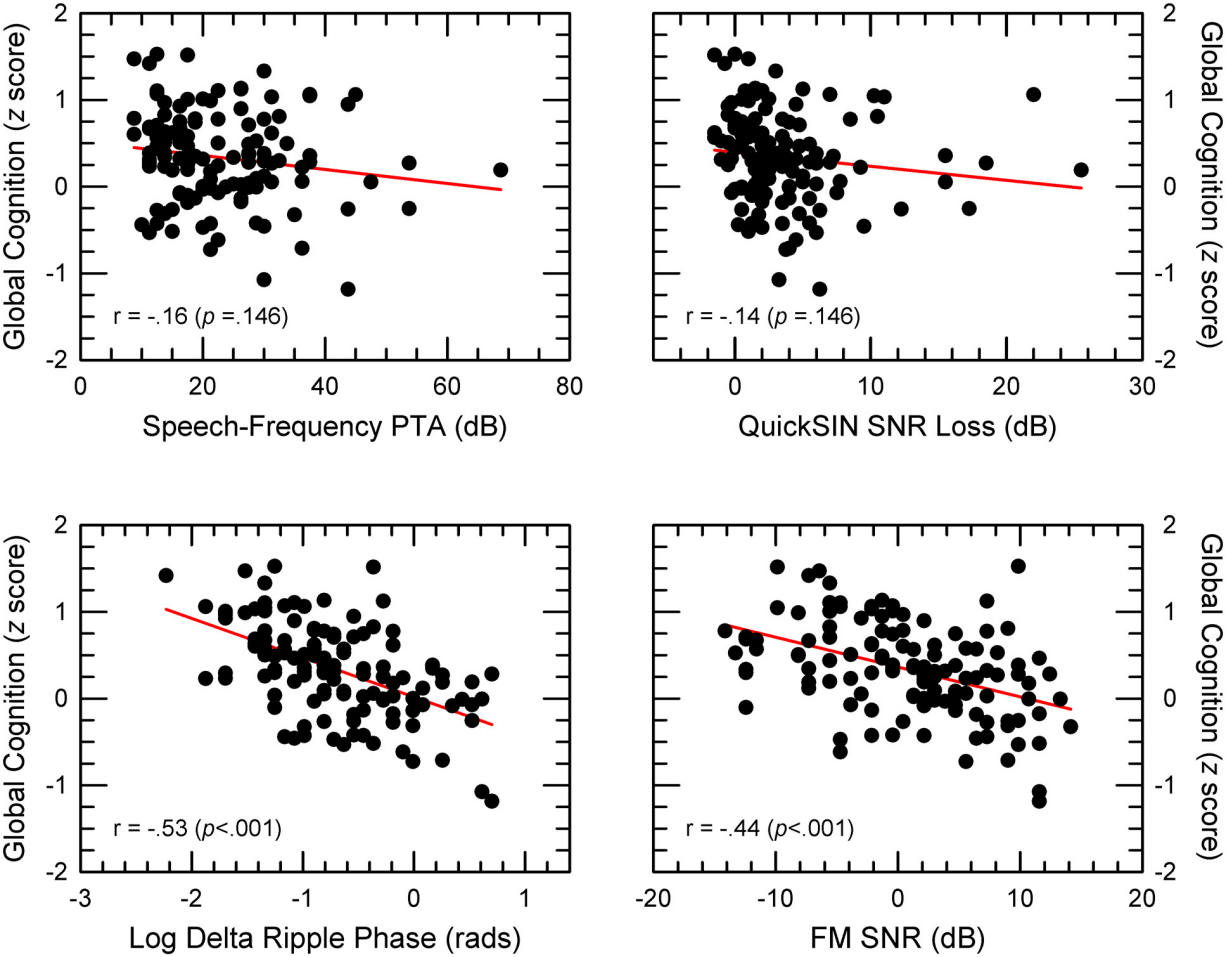
Relationships between global cognition and auditory abilities. In separate panels, individual global-cognition *z* scores as a function of each of the four auditory metrics. The solid red line is a linear regression of the data. Correlation, with the Bonferonni-Holm corrected *p* value in parentheses, is indicated in the lower left corner of each panel.

The relationship between global cognition and each of the auditory abilities is shown in separate panels of Fig 7. A significant correlation between global cognition and auditory ability was obtained only for the ripple-phase and FM SNR thresholds. From among these four correlation coefficients, four pairwise comparisons were conducted between each significant (i.e., ripple phase and FM SNR) and nonsignificant (i.e., PTA and QuickSIN) coefficient using the Steiger [46] *z* test for comparing dependent correlations. With correction for multiple comparisons, the four contrasts were significant (*p* < .05). Results thus indicate a difference between the measures of spectral-pattern discrimination and either hearing sensitivity or speech-in-noise ability in terms of association to global cognitive performance.

Further evaluation of the association between global cognition and the four measures of auditory ability was conducted with a multiple linear regression model that controlled for the demographic variables age, gender, race, and years of education. The regression model was statistically significant [*F*(8,123) = 12.08, *p* < .001], and as estimated by an adjusted R^2^ of .419, accounted for slightly over 40% of the variance in global cognition (Table 3). Among the demographic covariates, age, education and race significantly contributed to the model prediction. For the four auditory metrics, only ripple-phase and FM SNR performance were significant.

**Table 3:** Relation of global cognition to demographic and test variables.

Independent Variable	Estimate	*SE*	95% CI (lower/upper)	*β*	*p* value	Squared Semi-Partial Correlation	Squared Structure Coefficient
**Age**	-.284	.007	-.028/.000	-.164	.048	.019	.087
**Gender**	-.137	.114	-.363/.090	-.084	.235	.007	.026
**Education**	.027	.011	.005/.050	.174	.017	.028	.098
**Race**	-.360	.085	-.528/-.192	-.338	>.001	.085	.485
**PTA**	.002	.006	-.009/.014	.047	.689	>.001	.057
**QuickSIN**	-.005	.013	-.031/.021	-.043	.700	>.001	.041
**Ripple phase**	-.232	.075	-.380/-.084	-.273	.002	.045	.619
**FM SNR**	-.013	.006	-.026/-.001	-.172	.034	.022	.420

Table entries are the estimated coefficient, standard error (*SE*), the lower and upper 95% confidence interval (CI) for the estimate, standardized coefficient (β), *p* value, squared semi-partial correlation, and squared structure coefficient of a linear regression model predicting global cognition. For the model, *F*(8,123) = 12.08, *p* < .001, and R^2^ and adjusted R^2^ were .457 and.419, respectively.

The squared structure coefficients (the square of the bivariate correlation between each auditory metric and predicted global cognition) showed that ripple-phase and FM SNR thresholds accounted for 61.9 and 42.0%, respectively, of the variance in the regression model of global cognition. However, as indexed by the squared semi-partial correlations, the proportion of variance in global cognition uniquely explained by these variables was low. The squared semi-partial correlation for ripple-phase performance was .045, and .022 for FM SNR thresholds. These values are much lower than the corresponding squared correlations between global and either ripple-phase (.283) or FM SNR thresholds (.192). The low values of the squared semi-partial correlations indicate shared variance among the variables in the model, suggesting that some of the predictive power of the ripple-phase and FM SNR measures was not unique but shared with other covariates. Expressed as percent of variance explained by the model, the sum of the squared structure coefficients for all variables exceeded 100%, further indicating shared variance across covariates.

### Contributions of demographic characteristics and auditory abilities to global cognitive performance

To further explore the contributions of auditory and demographic measures to the prediction of global cognitive function, commonality analysis was used to partition the multiple regression effect described in Table 3 in terms of unique and shared variance. The unique contributions from the five significant covariates of the model (age, education, race, ripple phase, and FM SNR) summed to account for 43.6% of the variance (Table 4). The largest unique contribution was from race (18.7%), with ripple-phase and FM SNR thresholds accounting for 10.0 and 4.7%, respectively, of the model prediction. Across all combinations of variables, the largest contributions of shared variance involved the covariates race and ripple-phase and FM SNR thresholds. This shared variance between race and auditory spectral discrimination was not due to interactions. Added terms in the regression model for the interactions between race and the covariates ripple-phase and FM SNR thresholds were not significant. In contrast to the results for the two measures of spectral discrimination, the shared variance from either speech-frequency PTA or QuickSIN SNR Loss with race was quite low, as was the shared variance of these two metrics with either measure of spectral discrimination. In all cases, these shared variances involving either hearing sensitivity or speech-in-noise ability accounted for less than 1% of the variance in the regression model of global cognition.

**Table 4:** Unique and shared variance components in predicting global cognition.

Component	Variance explained (%)
**Unique: race**	18.7
**Shared: race, ripple phase, FM SNR**	17.4
**Shared: race, ripple phase**	16.3
**Unique: ripple phase**	10.0
**Shared: ripple phase, FM SNR**	6.6
**Unique: education**	6.1
**Unique: FM SNR**	4.7
**Shared: race, FM SNR**	4.4
**Unique: age**	4.1

Unique and shared variance components contributing at least 4% to variance explained in the linear regression model of global cognition in Table 3.

### Grouping of auditory and cognitive abilities

Finally, the interrelationships among auditory and cognitive abilities and subject age were examined using factor analysis. The five domain-specific cognitive *z* scores (episodic, semantic, and working memory, perceptual speed, and visuospatial abilities) along with the four auditory thresholds (PTA, QuickSIN SNR Loss, ripple-phase and FM SNR thresholds) and subject age were submitted to a principal-components factor analysis using an eigenvalue < 1 stopping rule. Three factors emerged, accounting for 66.8% of the total variance. The Kaiser-Meyer-Olkin sampling adequacy was .78, with Bartlett’s test of sphericity significant (*p* < .001). Communalities, the proportion of variance explained by the derived factors, across the nine variables ranged from .51 to .84. Anticipating correlations among components, oblique rotation (Oblimin with Kaiser Normalization) was used. Table 5 shows the component weights from the pattern matrix for the three-factor solution. The pattern of component weightings separated the auditory variables, with speech-frequency PTA and speech-in-noise ability loading almost exclusively on component 2 and the two measures of spectral-pattern discrimination on component 3. Among domain-specific cognitive metrics, processing speed and episodic and semantic memory loaded primarily on component 1. Working memory showed largest weighting on component 3, joining the auditory measures of spectral-pattern discrimination, with the weighting of visuospatial abilities almost evenly split between components 1 and 3.

**Table 5:** Pattern matrix.

		Component	
	1	2	3
**Age**	**-.451**	**.659**	**-.359**
**Semantic memory**	**.858**	.110	-.064
**Episodic memory**	**.777**	.057	-.051
**Perceptual speed**	**.720**	-.158	-.114
**Visuospatial abilities**	**.441**	.100	**-.434**
**Working memory**	.037	.114	**-.757**
**Speech-frequency PTA**	.101	**.912**	.158
**QuickSIN SNR Loss**	.096	**.903**	.105
**Ripple-phase threshold**	-.154	.230	**.667**
**FM SNR threshold**	-.077	.196	**.639**

Component weighting of age, cognitive, and auditory variables obtained in a principal-components factor analysis using Oblimin rotation with Kaiser Normalization. Magnitude of component weights > 0.3 are highlighted via bold font and underscore.

The results from both the regression modeling and factor analysis indicated low associations between speech-in-noise thresholds and cognitive abilities. In a review of studies evaluating the association between speech reception and cognitive ability, Akeroyd [47] found that tests of working memory had mostly given significant results. To confirm the pattern of component weighting of working memory in the factor analysis, three separate linear regression models were run with working memory as the independent variable and either ripple-phase threshold, FM SNR threshold, or QuickSIN SNR Loss as the dependent variable (Table 6). The squared correlations between the working memory score and each dependent variable were .135, .110, and .002 for ripple-phase, FM SNR, and QuickSIN results, respectively. When controlling for the demographic variables age, gender, race, and years of education, working memory was significantly associated with both measures of spectral-pattern discrimination, but not with speech-in-noise ability. The squared structure coefficients indicated that working memory scores accounted for 49.2 and 53.0% of the variance in the regression model of ripple-phase and FM SNR thresholds, respectively.

**Table 6:** Relation of spectral-pattern discrimination and speech-in-noise ability to working memory.

Model	Dependent Variable	Estimate	*SE*	95% CI (lower/upper)	*β*	*p* value	Squared Semi-Partial Correlation	Squared Structure Coefficient
**1**	**Ripple phase**	-.183	.064	-.309/-.057	-.247	.005	.051	.492
**2**	**FM SNR**	-2.063	.724	-3.496/-.629	-.256	.005	.055	.530
**3**	**QuickSIN**	-.741	.468	-1.668/.185	-.139	.116	.016	.011

From three separate linear regression models that controlled for age, gender, race, and education, table entries are the estimated coefficient, standard error (*SE*), the lower and upper 95% confidence interval (CI) for the estimate, standardized coefficient (β), *p* value, squared semi-partial correlation, and squared structure coefficient for the independent variable working memory. For model 1, *F*(5,123) = 8.88, *p* < .001; model 2, *F*(5,123) = 6.18, *p* < .001; and model 3, *F*(6,123) = 7.90, *p* < .001. R^2^ (adjusted R^2^) was .273 (.243), .208 (.174), and .251 (.219) for models 1, 2, and 3, respectively.

As in the analysis of global cognition, the squared semi-partial correlations, in this case indicating the proportion of variance uniquely explained by working memory, were low due to shared variance. The squared semi-partial correlation of the independent variable working memory to the dependent variable ripple-phase threshold was .051, and .055 for the dependent variable FM SNR threshold (Table 6). Partitioning of each regression effect through commonality analysis revealed that the largest contributions of shared variance to the predictive power of the variable working memory in the models involved the covariate race. The shared variance between race and working memory was 31.2 and 29.7% of the regression-model variance for ripple-phase and FM SNR thresholds, respectively.

## Discussion

The goal of this study was to evaluate the contributions of both peripheral and central auditory abilities to measures of cognitive status in older adults. The cohort contained roughly equal numbers of Black and White subjects without known dementia. Audiometric results as assessed by the speech-frequency PTA indicated age-appropriate extent of hearing loss [30,48]. There was a trend for slightly better hearing sensitivity among Black than White subjects, with the 2.5-dB group difference not significant. Lin et al. [30] reported a significant effect of race based on a larger PTA difference of 5.8 dB. Difference in test setting may have contributed to the difference in result between studies. In the current protocol, testing in a home environment may have increased the variability in the detection threshold measures compared to that obtained in the sound-isolating room used in the work of Lin et al.

Along with audiometric results, auditory abilities were assessed with a speech-in-noise intelligibility threshold and two measures of the ability to discriminate either static or dynamic spectral patterns. Only the two discrimination metrics significantly contributed in regression modeling of cognitive performance. This absence of significant association between hearing sensitivity and cognitive function is consistent with some [6–11] but not all [1–5] past studies of older adults. Again, audiometric protocol may have affected current results. Alternatively, restricting subject enrollment to those without known dementia along with the use of a 12-test battery for cognitive evaluation may have provided a more accurate estimation of associations for the specific cohort of the current study. Though contribution of only the spectral-pattern discrimination metrics to prediction of cognitive function does not support either the common-cause or cascading-consequence hypothesis linking deficits in hearing sensitivity to cognitive status, it may indicate involvement of age-related deficits in temporal encoding of suprathreshold sensory information as suggested by Humes et al. [11].

Results failed to show significant association between speech-in-noise ability as assessed by QuickSIN and either a summary metric of global cognition or working memory. In past work, many [47,49–52] but not all [10,53–55] studies have reported a significant association between speech perception and cognitive performance in older listeners. Across studies, variation in the strength of association is at least in part attributable to methodological differences, including speech materials, presence and type of speech masking, task requirements (e.g., identification or comprehension), use of amplification to compensate for hearing loss, cognitive tests used, and cognitive status of study participants. Furthermore, Humes [56] argued that evidence of significant involvement of cognitive factors in speech processing by older adults is best obtained with processing that ensures speech audibility across the stimulus frequency spectrum, a manipulation not used in the home-based testing of the current work.

A common finding among many of the studies of speech perception cited above was a strong association for older listeners between degree of hearing loss and speech abilities. The factor analysis of the current work showed this association through the grouping of age, speech-frequency PTA, and QuickSIN SNR Loss as the dominant variables of a common factor. The two remaining auditory variables assessing ability to discriminate spectral patterns grouped on a separate factor along with the cognitive measures of working memory and visuospatial abilities. This pattern of factor weighting is consistent with the findings from the regression modeling which found significant association between both measures of spectral-pattern discrimination and a summary metric of global cognition.

Working memory is commonly considered to show involvement of short-term memory and subsequent information processing [57–58]. Our working memory measures were adapted from the Revised Wechsler Memory Scale [38], which represents a clinical measurement benchmark. The forward digit span test is a “simple” span task, while the backward digit span task involves both temporary storage and manipulation of digits in memory. In the review of Akeroyd [47], the most consistent associations between speech scores and working memory were found with the reading span task. A “complex” span task, the reading span test requires that participants appraise the semantic coherence of each sentence while maintaining the first and last words of each sentence in memory. While some past work has shown strong correlation between simple and complex span tasks [34] with indication from other work that both measure the same basic subcomponent processes [59], recent meta-analysis of procedures used to assess working memory supports the view that different tests can tap largely separate components of working memory [60]. Thus, it is possible that the complexity of the reading span task allows for a closer association to speech perception than we observed with the forward and backward digit span task. Several recent studies, however, have not confirmed independent association between reading span performance and speech abilities [61–63]. Further study is warranted, and we therefore do not interpret our results obtained with the forward and backward digit span tasks as encompassing all aspects of processing assessed in other tests of working memory.

In a recent study of normal-hearing older adults, Füllgrabe et al. [63] evaluated auditory temporal processing in terms of both envelope and fine-structure cues, along with a variety of cognitive measures. Results showed significant moderate correlations between temporal fine-structure sensitivity and performance on several cognitive tests, including forward digit span and other measures of working memory. Fogerty et al. [64] evaluated the contribution of cognitive measures from WAIS-III [65] to predictions of auditory temporal-order processing ability with vowel stimuli. For older listeners with monaural two-vowel stimuli, 29% of the variance in performance was accounted for by cognitive status. With increasing stimulus length to four vowels, less than 4% of the variance was predicted by cognitive performance. In the current study, without consideration of covariates, 28% of the variance in ripple-phase discrimination was accounted for by the measure of global cognition with the value dropping to 20% for FM SNR thresholds. Since stimulus duration increased with number of vowels in the work of Fogerty et al. [64], either factor (stimulus duration or number of sequence events) could underlie the change in association between cognitive performance and the ability to identify sequence order. Stimulus duration, however, was the same (500 ms) for the two conditions evaluating spectral-pattern discrimination. Thus, current results suggest a trend in which the association between cognitive status and auditory processing of complex stimuli diminishes in tasks requiring that listeners monitor multiple changes that occur across a fixed stimulus duration.

With inclusion of demographic covariates in regression modeling, ripple-phase and FM SNR thresholds uniquely accounted for only 10.0 and 4.7%, respectively, of the model prediction. Commonality analysis indicated that the drop from values of 28 and 20% was primarily due to a high degree of shared variance with the covariate race. Furthermore, White subjects showed significantly better performance than Black subjects in the two discrimination tasks. A possible functional explanation for these effects is that Black subjects, more so than Whites, performed like high-threshold or low-risk observers who had a reluctance to incorporate partial or uncertain information into their response. A consequence of this approach is a steepening of the slope of the psychometric function and an elevation in thresholds. While this explanation does not offer a basis for the adopted listening strategy, presumably factors similar to those that underlie racial differences on cognitive measures [23–28] could also bias listening strategy. The current psychoacoustic protocol assessed performance with 42 trials spread across six levels of the independent variable. Consequently, the slope of the psychometric functions could not be reliably determined. Evaluation of adopted listening strategy is a goal of future work.

Alternatively, the better performance of White than Black subjects in the auditory spectral-discrimination tasks may reflect racial differences in performance on tests of working memory. Auditory discrimination tasks that require comparison of stimuli across multiple observation intervals must inherently include some level of involvement of working memory. Consequently, the reduced working-memory ability of Black compared to White subjects could lead to poorer performance in the auditory discrimination tasks. Though this argument makes no assumption regarding the basis for differences in working memory, it implies an effect in direction opposite to a cascading-consequences approach. Specifically the suggestion is that working memory affects measurement of auditory processing, rather than vice versa. However, this does not necessarily mean that working memory affects the underlying auditory processing, with effect limited to the psychophysical assessment of the processing.

The motivation for evaluation of the association between cognitive status and results of tests of static and dynamic spectral-pattern discrimination was to avoid the confounding effect of cognitive involvement in central auditory measures reliant on speech processing. The involvement of working memory in the metrics of auditory spectral-pattern discrimination potentially represents a similar confound. Despite working memory being a significant predictor of the ripple-phase and FM SNR thresholds in separate regression models, the variance uniquely explained by working memory as estimated by the squared semi-partial correlation was roughly only 5% in each case. Thus, while showing involvement of cognitive function in the two metrics of auditory spectral-pattern discrimination, we interpret the psychoacoustic results primarily as measures of aspects of central auditory processing.

The study was designed to incorporate several brief tests of auditory abilities into a much larger protocol evaluating participants of two ongoing studies of aging and cognition, with all testing at the participant’s home or residence. This design led to several limitations. Due to concern with the reliability of high-frequency testing in a home environment, audiometric testing did not go above 4 kHz. Though commonly assessing hearing sensitivity above 4 kHz, most past studies have not incorporated high-frequency sensitivity into the reported metrics used to determine the association between hearing acuity and cognitive status. Nevertheless, information on high-frequency hearing would be useful and will be included in future work. Audiometric testing did not include measurement of bone-conduction thresholds or tympanometry. Also, the hearing history of study participants was not available. These factors limited our ability to consider results in terms of the etiology of a hearing loss when present. Finally, measurement of spectral-pattern discrimination was based on a single 42-trial block. Consequently, we know neither the extent of within-subject variability for these measures nor the possible effect of additional training on performance.

In summary, the current work incorporated into home-based testing two measures of central auditory ability based on spectral pattern discrimination, along with assessment of hearing sensitivity and speech-in-noise perception. For a cohort of older adults without diagnosis of dementia, neither hearing thresholds nor speech-in-noise ability showed significant association to a summary measure of global cognition. Using auditory metrics that avoided the confounding effect of speech materials, the two measures of spectral-pattern discrimination, however, were significantly associated with cognitive performance. Among cognitive measures, factor analysis showed that working memory and visuospatial abilities had the strongest interrelationship to spectral-pattern discrimination. Regression analysis indicated substantial shared variance among the covariates race and both spectral discrimination measures in accounting for cognitive performance, with possible basis in either the decision strategy used by observers or differences in working memory affecting threshold measurement in the auditory tasks.
